# Book Review: International Perspectives on CLIL

**DOI:** 10.3389/fpsyg.2021.737301

**Published:** 2021-08-17

**Authors:** Xiaolan He, Zhiwu Zhang

**Affiliations:** ^1^Public Foreign Language Teaching Department, Hunan First Normal University, Changsha, China; ^2^School of Foreign Studies, Changsha University of Science and Technology, Changsha, China

**Keywords:** multilingualism, bilingualism, CLIL, professional development, content and language integrated learning

Despite its origin as a product of Europe's multilingual policy in the mid-1990's, Content and Language Integrated Learning (CLIL) has arrested worldwide attention for its methodological value and pedagogical innovations in the learning of second or foreign languages alongside content subjects (Bellés-Fortuño, 2021). Globally, local practices of CLIL continue to be implemented through personal choices of grassroots stakeholders, and vary in accordance with its specific educational context (Van Kampen et al., 2020), featuring variants on a continuum of language-driven CLIL and content-driven CLIL (Ball et al., 2015). This edited volume presents an encompassing exploration of the concrete culturally-situated, locally-adapted practice models in CLIL implementation and CLIL professional development in diverse geographical and educational contexts, boasting theoretical and pedagogical insights for practitioners, researchers and teacher educators.

This volume consists of 14 chapters. Besides an overview of CLIL in Chapter 1 and its current status and future in Chapter 14, the rest is grouped into two thematic parts, which are key to successful CLIL implementation: CLIL practices (Chapters 2–7) and CLIL professional development and awareness (Chapters 8–13). The first part covers such topics as authenticity and motivation, assessment, translanguaging, essential questions, and intercultural competence development while the second addresses the “who and how” in CLIL implementation, probing such topics: teacher training needs, teacher preparation, teaching materials development and evaluation, which lays the foundation for the enhancement of practice for CLIL teachers (Banegas and del Pozo Beamud, 2020).

As a timely addition to the literature in the under-explored areas in CLIL, an exploration of local experiences integrating theory and practice, and a window to the global insights about current CLIL implementation, it is a worthy recommendation for the following reasons.

Firstly, it stands out for its extensive elaboration on the two indispensable parts of successful CLIL implementation. Researchers have traditionally dedicated themselves to the study of solutions, advice, and models for effective and efficient CLIL implementation in the classroom (Dallinger et al., 2016), but literature related to CLIL teacher education or teacher training programs is not sufficient (Bellés-Fortuño, 2021). This practice-oriented volume, with its well-chosen contributions on CLIL practices and CLIL professional development, distinguishes itself from previous researches by drawing together recent international research to offer thought-provoking practical guidance in both areas to those interested in designing and implementing CLIL in their own educational settings.

Secondly, besides the traditional sociocultural theory and systematic functional linguistics underpinning CLIL, the volume integrates a diversity of new theoretical perspectives with CLIL practice, such as the 4C's framework (Cognition, Content, Communication, Culture) (Chapters 1 and 12), the Cognitive Discourse Functions model (Chapter 3), and translanguaging (Chapter 5). These approaches provide new interpretations of those local and context-responsive CLIL implementations, expanding our understandings from the confinement of content-driven CLIL in European settings to a diversity of CLIL variants worldwide. For example, in Chapter 5, Garzón-Díaz analyzes how L2 low achievers in a Colombian public school develop scientific literacy through the use of both L1 and L2. It is indicated that translanguaging may play a key role in helping learners construct knowledge in bilingual or multilingual contexts.

Thirdly, even though the edited volume by Carrió-Pastor and Bellés-Fortuño (2021) has dedicated half of its content to topics related to CLIL pedagogy in multicultural and multilingual classrooms, this volume, by accommodating various CLIL initiatives in different educational settings and geographical contexts, fills the gap between CLIL theory and CLIL practice by highlighting CLIL teaching practices and constitutes a good addition for the pedagogical community with CLIL practice insights, providing an updated view of the whole CLIL educational scenario around the globe. For instance, how CLIL is practiced in classrooms worldwide is illustrated with examples from different countries, such as Spain (Chapter 3), Japan (Chapters 4 and 6), and Austria (Chapter 10). Readers can not only gain an updated view of international CLIL practices in this field geographically, they can also engage with views and experiences from different educational levels and settings, for instance, primary level (Chapter 12), secondary level (Chapters 3 and 5), and tertiary level (Chapters 2 and 4); pre-service program (Chapters 8 and 9) and in-service teacher program (Chapters 10 and 11). These accounts of what has worked and what has not can enable practitioners, researchers, and administrators to understand how learners develop subject-matter knowledge and linguistic skills in an additional language system through CLIL (Pérez Cañado, 2020).

Finally, two sections in each of the body chapters, namely, “Suggested Further Reading” (a concise recommendation of updated or classical readings) and “Engaging Priorities” (a list of questions to guide readers to explore further in their own contexts), are purposefully provided to inspire readers to explore in their respective contexts.

Overall, this volume takes a global stance by a careful selection of contributions from well-established international researchers on CLIL teaching and learning practices, centering around how CLIL is implemented, how localized CLIL practice models are developed, and their pedagogical implications. The CLIL landscapes portrayed in various geographical and educational settings inspire students, teachers, researchers, and administrators about pedagogical practices and professional development. The suggested readings and engaging priorities will leave readers with insightful resources to probe on. We find this volume a highly recommended reading, and it will appeal to those interested in understanding empirical and theoretical forefronts in CLIL and will shed light on English language teaching, teacher professional development as well as bilingual or multilingual education.

## Author Contributions

XH and ZZ co-selected the book, drafted the review, and engaged in analysis and revision. Both have approved it for publication.

## Conflict of Interest

The authors declare that the research was conducted in the absence of any commercial or financial relationships that could be construed as a potential conflict of interest.

## Publisher's Note

All claims expressed in this article are solely those of the authors and do not necessarily represent those of their affiliated organizations, or those of the publisher, the editors and the reviewers. Any product that may be evaluated in this article, or claim that may be made by its manufacturer, is not guaranteed or endorsed by the publisher.
